# Evaluation of epithelial progenitor cells and growth factors in a preclinical model of wound healing induced by mesenchymal stromal cells

**DOI:** 10.1042/BSR20200461

**Published:** 2020-07-22

**Authors:** Giselle Ramos-Gonzalez, Olga Wittig, Dylana Diaz-Solano, Lianeth Salazar, Carlos Ayala-Grosso, Jose E. Cardier

**Affiliations:** 1 Unidad de Terapia Celular - Laboratorio de Patología Celular y Molecular, Instituto Venezolano de Investigaciones Científicas (IVIC), Apartado 21827, Caracas 1020-A, Venezuela; 2 Servicio de Cirugía Plastica, Hospital de la Cruz Roja, Caracas 1080, Venezuela

**Keywords:** EPC, FGF, KGF, MSC, re-epithelialization, wound healing

## Abstract

**Background:** Skin wounds continue to be a global health problem. Several cellular therapy protocols have been used to improve and accelerate skin wound healing. Here, we evaluated the effect of transplantation of mesenchymal stromal cells (MSC) on the wound re-epithelialization process and its possible relationship with the presence of epithelial progenitor cells (EPC) and the expression of growth factors. **Methods:** An experimental wound model was developed in C57BL/6 mice. Human MSCs seeded on collagen membranes (CM) were implanted on wounds. As controls, animals with wounds without treatment or treated with CM were established. Histological and immunohistochemical (IH) studies were performed at day 3 post-treatment to detect early skin wound changes associated with the presence of EPC expressing Lgr6 and CD34 markers and the expression of keratinocyte growth factor (KGF) and basic fibroblast growth factor (bFGF). **Results:** MSC transplantation enhanced skin wound re-epithelialization, as compared with controls. It was associated with an increase in Lgr6^+^ and CD34^+^ cells and the expression of KGF and bFGF in the wound bed. **Conclusion:** Our results show that cutaneous wound healing induced by MSC is associated with an increase in EPC and growth factors. These preclinical results support the possible clinical use of MSC to treat cutaneous wounds.

## Introduction

Cutaneous wound healing comprises several stages; one of them is the re-epithelialization, which is fundamental for tissue wound repair [1] It initiates with the activation, migration and proliferation of epithelial progenitor cells (EPC) located in the interfollicular epidermis (IFE) and hair follicles (HFs) [2]. In addition, several growth factors (including keratinocyte growth factor (KGF) and basic fibroblast growth factor (bFGF)) and cytokines participate in the repair process by promoting proliferation, differentiation and migration of EPC from healthy skin to the wound center [3].

Although numerous treatments have been applied for improving wound healing, it continues being a serious medical problem in many patients. Recently, cellular therapy has been proposed for inducing skin wound repair [4]. Numerous studies have focused on the use of mesenchymal stromal cells (MSC) transplantation for inducing wound repair [5–8].

MSC constitute a population of cells with a multipotential capacity of differentiation [9–11]. There is evidence showing that MSC participate in the process of wound healing [12–14]. It has been suggested that MSC may induce cutaneous wound repair by regulating the inflammatory and immune responses [15,16] These effects are mediated by paracrine signals generated from MSC [17,18], which may induce migration, proliferation and differentiation of skin EPC [3,19]. Importantly, due to the low expression of MHC class I and II and no expression of costimulatory molecules, the possibility of rejection in allogeneic or xenogeneic models of transplantation is very low [20,21]. Based on this knowledge, the use of MSC as a potential therapeutic strategy to induce cutaneous wound healing is highly considered in regenerative medicine. In this work, we used an animal skin wound model to evaluate the capacity of human MSC to induce wound healing and its possible relationship with the presence of EPC and the expression of growth factors.

## Materials and methods

### Animals

Male C57BL/6 mice (8-week-old) were obtained from the IVIC Laboratory Animal Center and maintained on a standard laboratory diet and housed in a controlled environment. All animal experimentation was performed at the IVIC following institutional guidelines and the National Institutes of Health guide for the care and use of laboratory animals. The study protocol was approved by the Animal Committee of IVIC (COBIANIM2014-04).

### Reagents and culture medium

FITC or phycoerythrin (PE)-conjugated monoclonal antibodies anti-human CD73 and CD90 were from BD Biosciences (Franklin Lakes, NJ, U.S.A.). Monoclonal antibodies anti-mouse CD34 were from Biolegend (San Diego, CA, U.S.A.). Polyclonal antibody anti-human Lgr6 was from Novus Biologicals (Littleton, CO, U.S.A.). Polyclonal antibodies anti-human KGF and bFGF) were from R&D Systems (Minneapolis, MN, U.S.A.). Atelocollagen membranes (CM) were purchased from Cosmo Bio (Tokyo, Japan). α-MEM medium was from Life Technologies (U.S.A.) and Chang medium was from Irvine Scientific (U.S.A.).

### Isolation and culture of bone marrow MSC

MSC used in this work were from healthy patients treated for bone regeneration, due to pseudarthrosis secondary to a fracture [22,23], who authorized the use of these cells by signing informed consent. These cells were from bone marrow and isolated from the posterior iliac crest of patients [22,23]. MSC were cultured, expanded and stored at −70°C until its use. For the present study, MSCs were thawed and expanded in αMEM-Chang medium until becoming near confluent.

### Phenotypic and functional analysis

The expression of MSC markers (CD73 and CD90) were evaluated by flow cytometry. Data collection and analysis of the fluorescent intensities were made using a BD Acurri™ C6 (Beckton Dickinson, U.S.A.). The multipotential capacity of MSC was examined following previous methodology using osteogenic, chondrogenic and adipogenic differentiation media [10,22,23].

### Transplantation of MSC on cutaneous wounds

**Figure 1:**
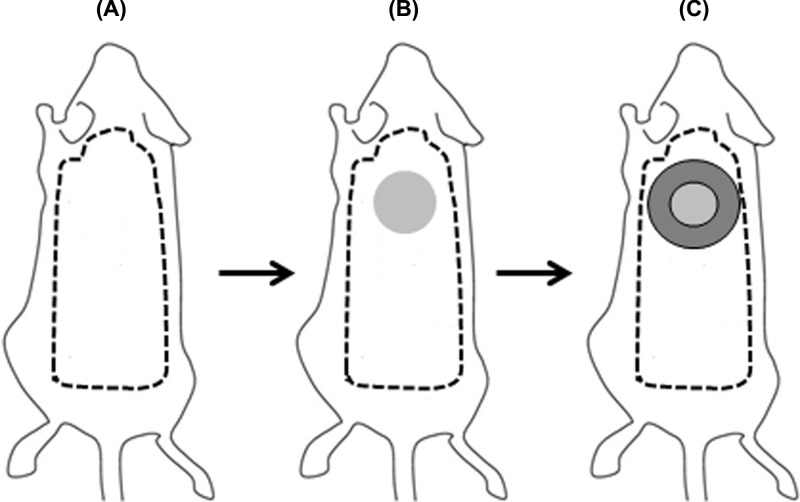
Schematic representation of the skin wound model C57BL/6 mice were anesthetized and hair was removed from the dorsal surface area (**A**). A 4-mm full thickness excisional skin wound was created on the dorsal area (**B**). A sterile band-aid was placed so that the wound was centered within it, and a transparent film dressing (Tegaderm 3M) was placed over the wound using an immediate-bonding adhesive to better fix the dressing to the skin (**C**).

MSC were seeded (3 × 10^5^ cells) on transwells containing CM for six-well culture plates (Atelo Cell, Cosmo Bio., Tokyo, Japan). The cells were maintained in culture until becoming near confluent. A skin wound model was performed in C57BL/6 mice. For this purpose, mice were anesthetized with a cocktail of Ketamine/Xylazine (100 mg/kg and 7.5 mg/kg body weight, respectively) and hair was removed from the dorsal surface area (Figure 1A). A 4-mm full thickness excisional skin wound was made on the dorsal area using a sterile disposable biopsy punch (Healthlink, Jacksonville, FL, U.S.A.) (Figure 1B). Inserts were removed from the plates and each CM containing MSC (MSC/CM) were cut in circles of approximately 4 mm in diameter and immediately applied on to the wound beds. A sterile band-aid was placed and centered over the wound. A transparent film dressing (Tegaderm 3M) was placed over the wound and an immediate-bonding adhesive was used to better fix the dressing to the skin (Figure 1C). As controls, groups of mice with wounds were uncovered or covered with CM without cells. The animals were housed individually. After 3 days of implantation, mice were killed by cervical dislocation and wounds were photographed. Wound area was determined before (d0) and after wounding (d3) by using ImageJ v1.48 (http://imagej.nih.gov/ij) program. It was expressed as the percentage of wound closure at day 3 as compared with d0 in each group (% of wound closure = [wound area d0 − wound area d3/area d0] × 100). Samples from the wounds were collected and included in paraffin for histological and immunohistochemical (IH) analysis. We evaluated early cellular changes occurring at day 3 after MSC transplantation because re-epithelialization of a skin wound of this size occurred at approximately day 7. Infiltrating polymorphonuclear cells were examined by two independent observers at low powered field microscopy. Data were reiterated by using a semiquantitative cross-scoring system (low = +, moderate = ++, high = +++).

### Histological and IH analysis of skin wound

Samples of skin wounds, including normal skin (NS), were fixed in 10% formalin solution, embedded in paraffin and sectioned at 4-µm-thick. Hematoxylin and Eosin (H&E) staining was performed. For immunostaining, paraffin sections were dewaxed, rehydrated and incubated with antibodies against Lgr6, CD34, KGF and bFGF.

### Wound analysis

Digital photographs from wounds were taken at days 0 and 3 post-wound. Percentage of re-epithelialization was determined using an analysis program (ImageJ). Percentage re-epithelialization was defined as the distance traveled by both epithelial tongues (long dn_1_ and long dn_2_) divided by the distance needed to travel to fully re-epithelialize the wound (long dn0) × 100: % re-epithelialization = [long dn_1_ + long dn_2_/long d0] × 100).

### Statistical analysis

Results are reported as mean ± standard error. We tested the data from the experiments for statistical significance using the Student’s *t* test for comparisons between groups. Differences were considered statistically significant at *P*≤0.05.

## Results

### Culture, phenotypical and functional characterization of MSC

**Figure 2:**
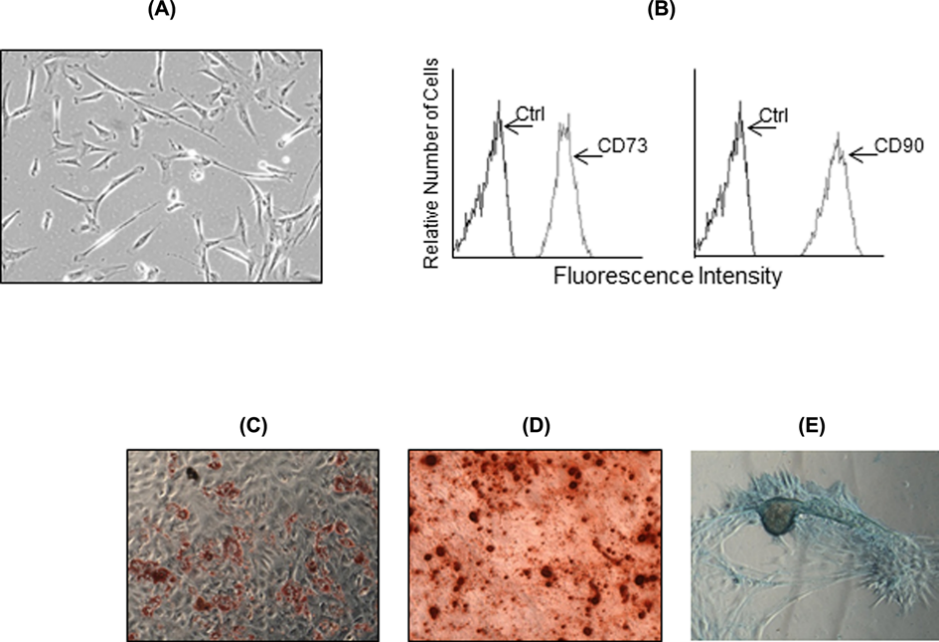
Phenotypical and functional characterization of MSC Microscopical observation shows the fibroblast-like morphology of MSC in culture (**A**). Flow cytometry analysis of MSC marker expression shows the expression of CD73 and CD90 (arrows). Negative controls were stained with the respective isotype (arrows) (**B**). Multipotent differentiation assays show the osteogenic (**C**), adipogenic (**D**) and chondrogenic (**E**) potential of MSC.

Cryopreserved MSC were thawed and cultured in α-MEM Chang medium. They showed fibroblast-like morphology in culture (Figure 2A) and expressed the typical MSC markers CD73 and CD90 (Figure 2B). By culturing in differentiation media, they showed their multipotential capacity of differentiation to adipogenic, osteogenic and chondrogenic cells (Figure 2C–E, respectively).

### Implant of MSC on cutaneous wounds

**Figure 3:**
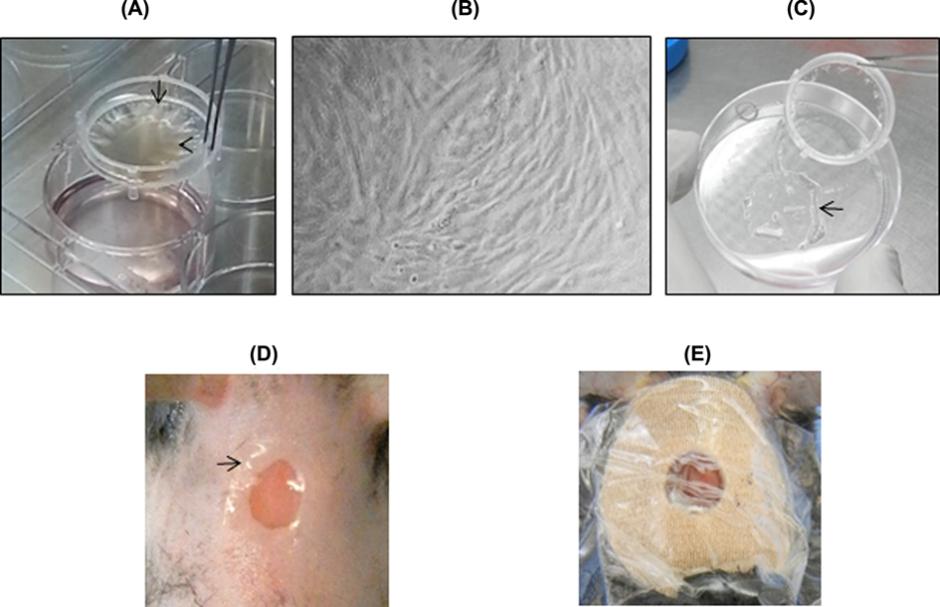
Implant of MSC on cutaneous wounds Culture medium containing MSC (head arrow, **A**) was added on CM transwell (arrow, A). After 72 h, MSC reached 100% confluence and exhibited fibroblast-like typical morphology on CM (**B**). MSC/CM were removed from the insert (arrow, **C**). CM (arrow) were cut and implanted on the bed of cutaneous wounds (**D**). The wound was covered with a band-aid and Tegaderm (**E**).

MSC were seeded on transwell inserts with CM (Figure 3A). After 72 h, cells grew reaching 100% confluence showing a fibroblastoid-like morphology on the CM (Figure 3B). MSC/CM were removed from the inserts and cut to the size of the wound (Figure 3C), and turned MSC side downward on to the wound bed (Figure 3D). The implanted MSC/CMs were in contact with the wound edges (Figure 3D). Finally, the wound was covered with a sterile band-aid and Tegaderm (Figure 3E).

### MSC promote early re-epithelialization of cutaneous wounds

**Figure 4:**
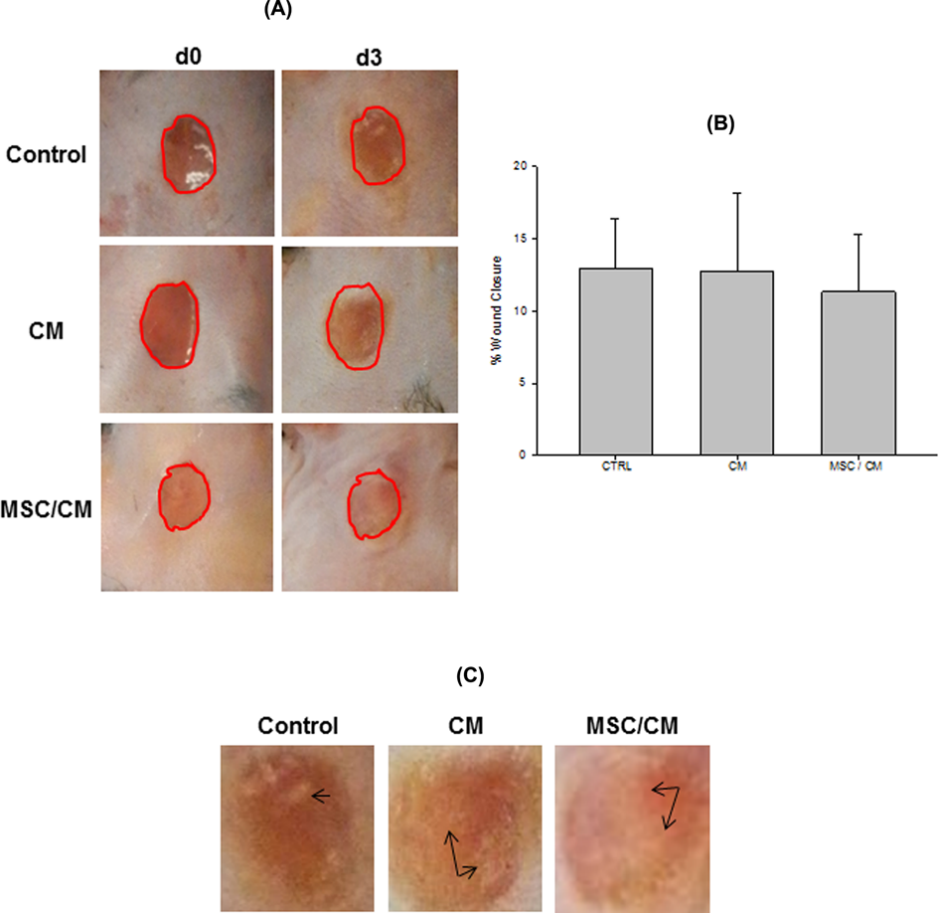
Evaluation of wound closure after MSC transplantation Wounds were evaluated before (d0) and after (d3) MSC transplantation. Wound closure was compared between the same experimental group (circle of the same size) (**A**). Wound closure was determined by using the ImageJ program. It was expressed as the percentage (means ± SE) of wound closure at day 3 as compared with d0 in each group (% of wound closure = [wound area d0 − wound area d3/area d0] × 100). There were not a statistically significant difference in wound closure between day 3 and d0 post-wounding in all groups (control, *n*=5; CM, *n*=5 and MSC/CM, *n*=4) (**B**). Signs of early re-epithelialization (whitish areas covering the wound surface) were observed in wounds (higher magnification) in all groups (**C**, arrows).

**Figure 5:**
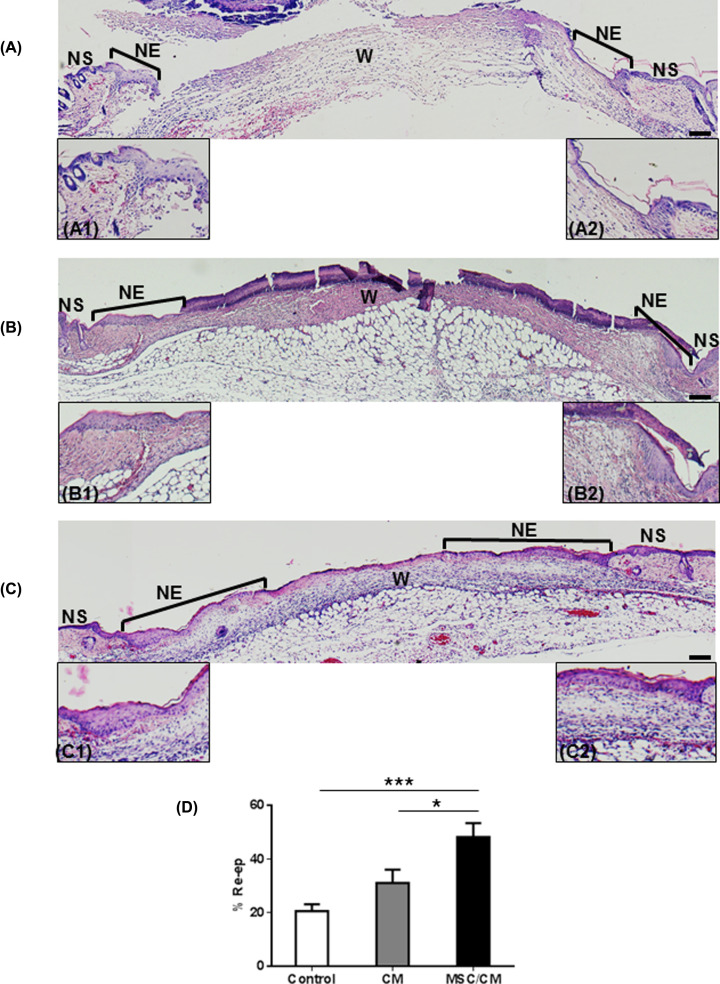
MSC transplantation enhances wound re-epithelialization Histological studies of wounds were performed in untreated wounds (control, **A**), CM-treated wounds (**B**) and MSC/CM-treated wounds (**C**), at day 3 post wounding. H&E-stained sections show NS and NE in the edges of wounds (W). Higher magnification of the newly formed epidermis is shown in each section (control, **A1**–**A2**; CM, **B1**–**B2**; and MSC/CM **C1**–**C2**). Histologic sections of wounds treated with MSC/CM show a larger area of re-epithelialization (C), as compared with those treated with CM alone (B) or control (A). Image analysis from histological sections show a significant increase in the percentage of re-epithelialization in wound treated with MSC/CM, as compared with control groups (**D**). Scale bar = 100 μm. Results are presented as means ± SE (Control, *n*=7; CM, *n*=7; MSC/CM, *n*=8). *P<0.05; ***P<0.001.

**Figure 6:**
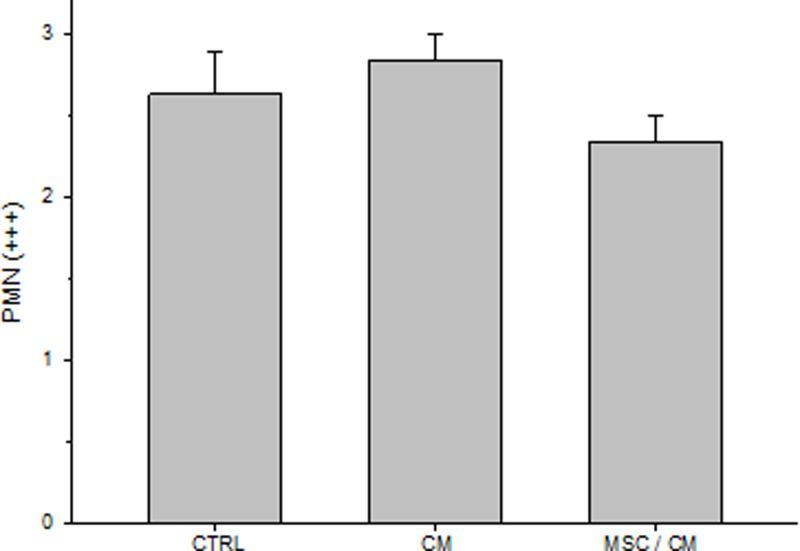
Evaluation of PMN in wounds PMN were evaluated before (d0) and after (d3) MSC implantation in control, MC- and MSC/MC-treated wounds. Analysis of PMN infiltrating wounds was performed by two independent observers (low powered field microscopy) by using a semiquantitative cross-scoring system (low = +, moderate = ++, high = +++). Results are presented as means ± SE. There was not a statistically significant difference in PMN infiltration between all experimental groups (control, *n*=8; CM, *n*=6; MSC/CM, *n*=9).

Because early cellular changes play a fundamental role in skin repair, we evaluated cutaneous wounds after 3 days of MSC implantation. For this purpose, animals were killed and wounds were evaluated. Macroscopic evaluation showed similar wound areas at day 0 and day 3 in each group (Figure 4A). Image analysis confirmed that there were not statistically significant difference in wound closure between day 3 and day 0 in all groups (Figure 4B). Signs of early re-epithelialization (whitish areas covering the wound surface) were observed in wounds from all groups (Figure 4C). However, they were more evident in the MSC/CM-treated group. The whole wound tissue, including NS, was collected and included in paraffin for histological analysis. Each sample was examined according to the presence of areas of NS, new epithelium (NE) and the wound area (W) (Figure 5). Histological studies showed small re-epithelialization areas (NE) in the periphery of wounds of control mice (non-treated) (Figure 5A). Similar results were observed in wounds implanted with CM alone (Figure 5B). In contrast, wounds treated with MSC/CM showed a larger re-epithelialization area from wound edge to the center of it (Figure 5C), as compared with those wounds treated with CM alone or without treatment (Figure 5A,B). These results were confirmed by using an image analysis software, which showed significant increases in re-epithelialization in wounds treated with MSC/CM, as compared with those treated with CM or control (Figure 5D). Epithelial thickening was observed in all groups, indicating the presence of hyperproliferative epidermis (Figure 5A–C). All wounds showed similar infiltration of PMN at day post-wounding (Figure 6).

### Detection of EPC expressing Lgr6 and CD34 in wounds treated with MSC

**Figure 7:**
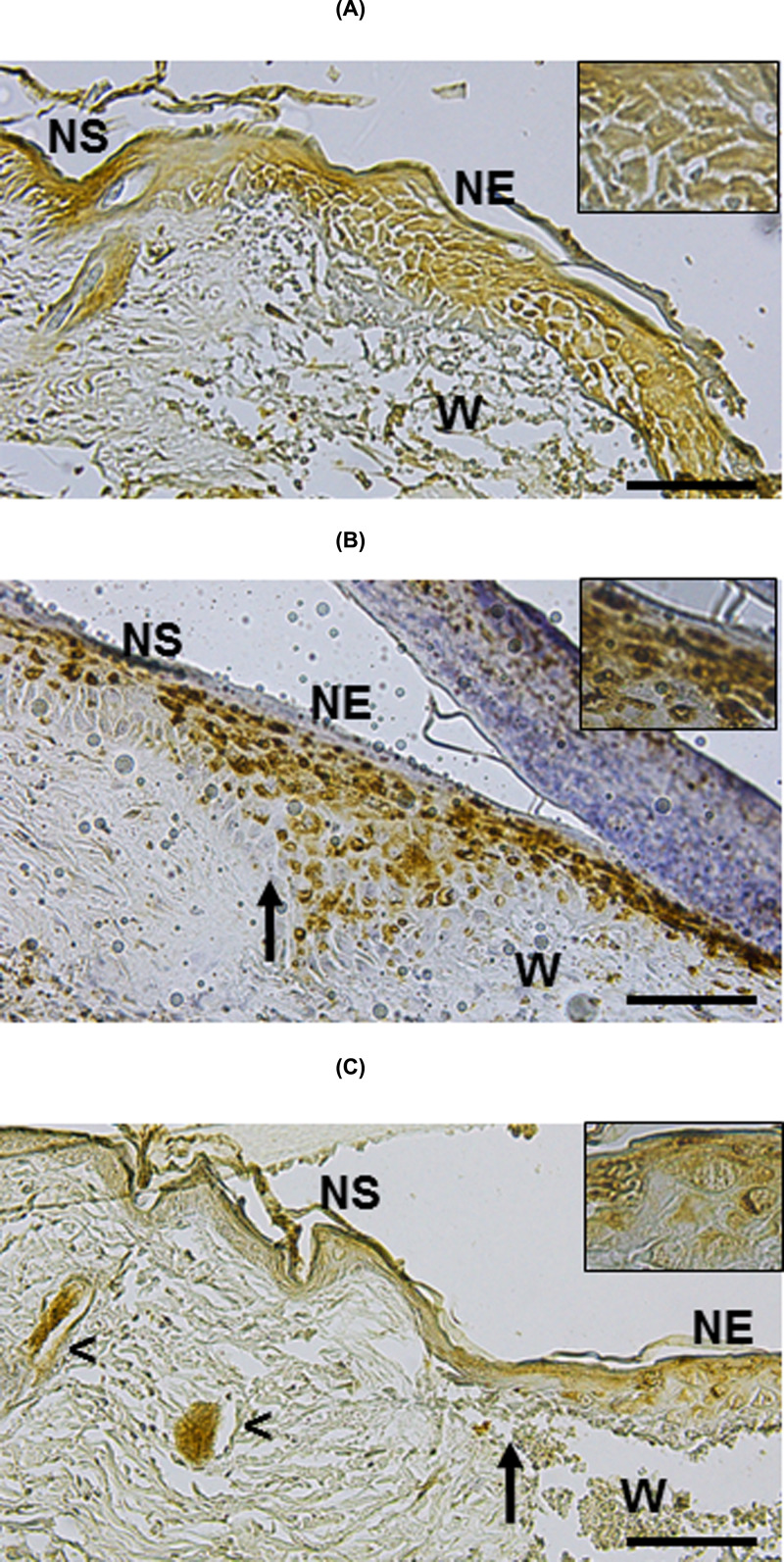
Detection of Lgr6^+^ progenitor cells in cutaneous wounds after 3 days of MSC implantation IH studies to detect Lgr6^+^ cells were performed in untreated wounds (control, **A**), CM-treated wounds (**B**) and MSC/CM-treated wounds (**C**). Tissue sections show NS and NE in the edges of wounds (W). Lgr6^+^ cells were present at the NS and NE of the control group and treated with CM (A,B, respectively). Most of the Lgr6^+^ cells were detected at the NE adjacent to the wound treated with MSC/CM (C). They were also detected in HFs close to the wound (head arrows). Each picture is representative of three different experiments, all with similar results. Scale bar = 50 μm.

**Figure 8:**
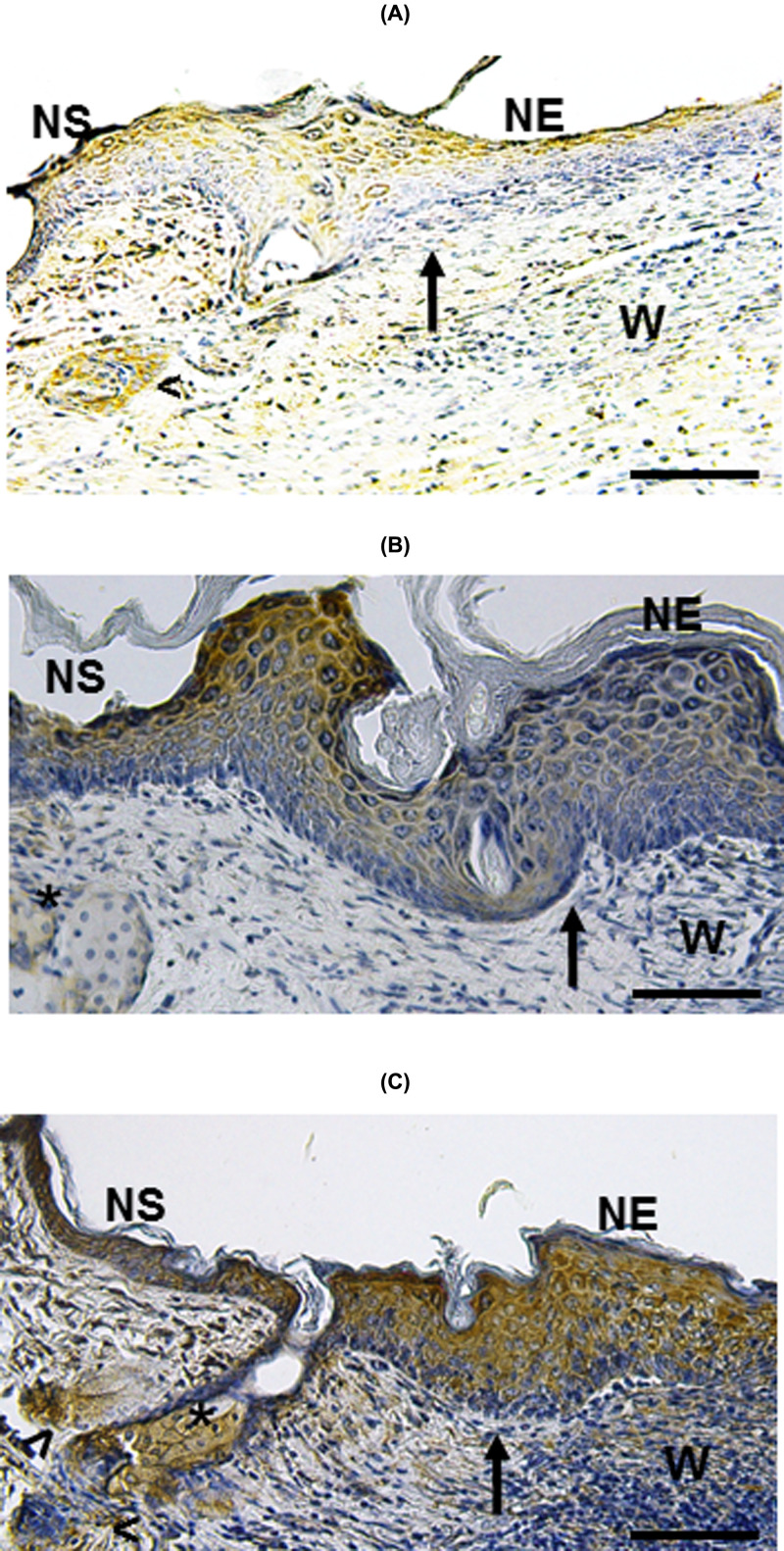
Detection of CD34^+^ cells in cutaneous wounds after 7 days of MSC implantation IH studies to detect CD34^+^ were performed in untreated wounds (control, **A**), CM-treated wounds (**B**) and MSC/CM-treated wounds (**C**). Tissue sections show NS and NE in the edges of wounds (W). A larger number of CD34^+^ cells are observed in epidermis adjacent to the wound, NE, HFs (head arrow) and SGs (asterisks) in the group treated MSC/CM (C), as compared with the control and the group treated with CM (A,B, respectively). Scale bar = 50 μm.

Based on evidence showing that EPC (Lgr6^+^ and CD34^+^ cells) are involved in skin wound repair [24], we evaluated whether re-epithelialization of wounds induced by MSC transplantation was associated with the presence of these cells. At day 3 post-MSC implant, IH evaluation showed Lgr6^+^ cells at the NS and NE of the control group and treated with CM (Figure 7A,B, respectively). In the group treated with MSC/CM, most of the Lgr6^+^ cells were detected in HFs and NE adjacent to the wound (Figure 7C). In contrast, few CD34^+^ cells were observed in all experimental groups at day 3 of evolution of the wound (not shown). However, higher number of CD34 cells was observed at day 7 post-treatment in NS, HFs, sebaceous gland (SGs) and NE adjacent to the wound (Figure 8C), as compared with the control and the group treated with CM (Figure 8A,B, respectively).

### Increased expression of KGF and bFGF in wounds treated with MSC

**Figure 9:**
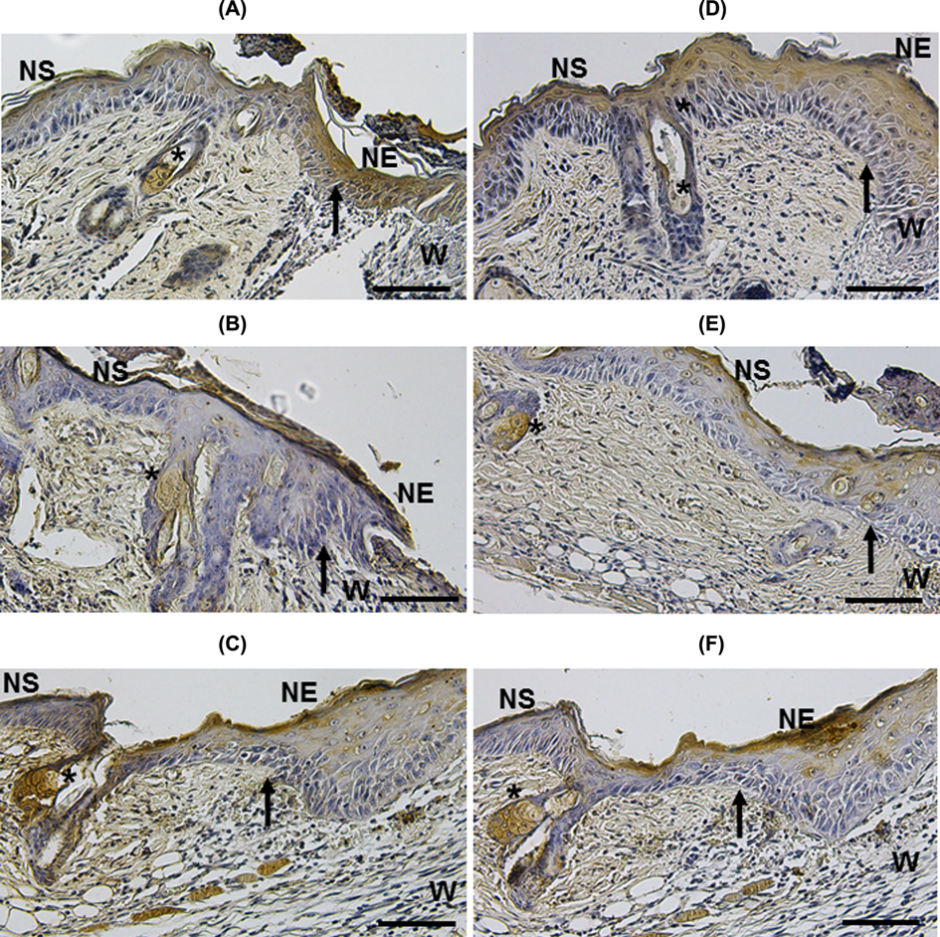
Increased expression of KGF and bFGF in cutaneous wounds after 3 days of MSC implantation Wounds treated with MSC/CM show an increased expression of KGF and bFGF (**C,F**, respectively) in NS, HF and SGs (asterisks), as compared with untreated (**A,D**, respectively) and CM-treated groups (**B,E**, respectively). Arrows indicate the edge of wound. Each picture is representative of three different experiments, all with similar results. Scale bar = 50 μm.

Because KGF and bFGF are expressed during the skin repair process [25], we evaluated whether re-epithelialization observed in wounds treated with MSC implantation was associated with increased expression of these factors. At day 3 post-MSC implant, IH analysis showed that wounds treated with MSC/CM had an increased expression of KGF (Figure 9C), as compared with untreated and CM-treated groups (Figure 9A,B, respectively). The increased expression of KGF was mainly observed in NS, HF and SGs (Figure 9C). Expression of bFGF was also augmented in wounds treated with MSC/CM (Figure 9F), as compared with untreated and CM-treated groups (Figure 9D,E, respectively). Similar to KGF, the increased expression of bFGF was observed in NS, HF and SGs (Figure 9F).

## Discussion

Numerous therapeutic procedures have been developed and applied to induce wound healing. However, many of them have failed to re-epithelialize injured skin. Recently, several experimental protocols based on cellular therapy have been used for inducing wound healing [26–28]. Here, we evaluate the capacity of MSC to induce cutaneous wound repair and its possible relationship with the presence of EPC and the expression of growth factors.

It is well known that MSC are involved in the biological process of skin wound repair [29–31]. It has been suggested that MSC may induce skin repair through paracrine signals and anti-inflammatory and immune regulatory effects [32–35]. Here, we used an experimental murine model to investigate the effect of human MSC on the re-epithelialization of full-thickness skin wound. Importantly, the possibility of rejection of human MSC, in this murine model, is very low because these cells have low immunogenicity [36–38]. In fact, we did not observe evidence of acute rejection and signs of an inflammatory reaction in wounds treated with human MSC.

Our results show histologically evidence that MSC enhance wound re-epithelialization as early as day 3 post-MSC implantation. Wounds treated with MSC showed larger re-epithelialization areas, from wound edge to the center of it, as compared with controls. It is known that the re-epithelialization process of skin wounds occurs by the proliferation, migration and differentiation of EPC located at different sites of healthy peripheral skin (i.e., epidermis, HF) [24,39–41]. Likewise, it is known that different EPC participates upon wounding. Recently, it has been reported that Lgr6^+^ cells located in the follicular isthmus, SG, IFE and follicular bulge participate in the process of wound repair [42,43]. In this work, we show that Lgr6+ cells were present in the three experimental groups. However, there were differences in the location of these cells between MSC/CM-treated wound and the control groups. Histologic evaluation showed Lgr6+ cells in the epidermis of NS and NE in control groups. In contrast, higher number of Lgr6+ cells was observed in NE and in HFs of wounds treated with MSC. Because there is evidence showing that Lgr6+ cells are primed to respond to wound signals in unwounded healthy skin [44], it is possible that paracrine signals from MSC induce a rapid mobilization of these cells, which result in higher re-epithelialization rate of wounds treated with these cells. On the other hand, few CD34+ EPCs were present in all experimental groups at early stages of the wound evolution (3 days). However, after 7 days, higher numbers of CD34+ cells were observed in NS, NE, HFs and SGs of wound treated with MSC. These results suggest a delayed response of CD34+ EPCs to paracrine signals from MSC. Together, our results support previous works showing that different EPC population participate at different stages of wound repair [44]. Our results suggest that Lgr6+ cells are the ones to respond first, and CD34^+^ cells would be the last to respond to paracrine signals from MSC for producing re-epithelialization of full-thickness wounds.

The wound re-epithelialization and repair process involves the expression of several growth factors [3]. Among them, it is known that KGF and bFGF participate in inducing skin wound re-epithelialization by regulating not only the proliferation and migration of EPC, but also of other cells (i.e., fibroblasts and endothelial cells) located in the periphery of the wounds [17,18,45]. In the present study we show that wounds treated with MSC had greater KGF and bFGF expression than those from the control groups. The high expression of KGF and bFGF may play an important role in inducing early re-epithelialization of wounds treated with MSC. The expression of these factors was mainly detected in NS, HF and SGs adjacent to the wound. The increased production of these growth factors could be due to paracrine signals from implanted MSC. However, other growth factors, produced as results of paracrine signals from transplanted MSC, may also participate in wound repair [17–46]. Interestingly, recent evidence has shown that paracrine signals from exosomes released by MSC may also contribute to the wound healing process [47–49].

In conclusion, our work shows that transplantation of MSC induces an early re-epithelialization of skin wounds, which is associated with an increase in EPC and epithelial growth factors. MSC loaded on CM may constitute a potential treatment for skin repair in cases of cutaneous wounds.

## Abbreviations

bFGF: basic fibroblast growth factor
CM: collagen membrane
EPC: epithelial progenitor cell
HF: hair follicle
IFE: interfollicular epidermis
IH: immunohistochemical
KGF: keratinocyte growth factor
MSC: mesenchymal stromal cell
MSC/CM: CM containing MSC
NE: new epithelium
NS: normal skin
PMN: polymorphonuclear
SG: sebaceous gland
